# Combined L-citrulline and glutathione supplementation increases the concentration of markers indicative of nitric oxide synthesis

**DOI:** 10.1186/s12970-015-0086-7

**Published:** 2015-06-10

**Authors:** Sarah McKinley-Barnard, Tom Andre, Masahiko Morita, Darryn S. Willoughby

**Affiliations:** Department of Health, Human Performance, and Recreation, Baylor University, Exercise and Biochemical Nutritional Lab, 76798 Waco, TX USA; Function Research Group, Healthcare Products Development Center, KYOWA HAKKO BIO CO., LTD., 2, Miyukigaoka, 305-0841 Tsukuba, Ibaraki Japan

**Keywords:** Nitric oxide, L-citrulline, L-arginine, Glutathione, Resistance exercise

## Abstract

**Background:**

Nitric oxide (NO) is endogenously synthesized from L-arginine and L-citrulline. Due to its effects on nitric oxide synthase (NOS), reduced glutathione (GSH) may protect against the oxidative reduction of NO. The present study determined the effectiveness of L-citrulline and/or GSH on markers indicative of NO synthesis in *in vivo* conditions with rodents and humans and also in an *in vitro* condition.

**Methods:**

In phase one, human umbilical vein endothelial cells (HUVECs) were treated with either 0.3 mM L-citrulline, 1 mM GSH (Setria®) or a combination of each at 0.3 mM. In phase two, Sprague–Dawley rats (8 weeks old) were randomly assigned to 3 groups and received either purified water, L-citrulline (500 mg/kg/day), or a combination of L-citrulline (500 mg/kg/day) and GSH (50 mg/kg/day) by oral gavage for 3 days. Blood samples were collected and plasma NOx (nitrite + nitrate) assessed. In phase three, resistance-trained males were randomly assigned to orally ingest either cellulose placebo (2.52 g/day), L-citrulline (2 g/day), GSH (1 g/day), or L-citrulline (2 g/day) + GSH (200 mg/day) for 7 days, and then perform a resistance exercise session involving 3 sets of 10-RM involving the elbow flexors. Venous blood was obtained and used to assess plasma cGMP, nitrite, and NOx.

**Results:**

In phase one, nitrite levels in cells treated with L-citrulline and GSH were significantly greater than control (*p* < 0.05). In phase two, plasma NOx with L-citrulline + GSH was significantly greater than control and L-citrulline (*p* < 0.05). In phase three, plasma cGMP was increased, but not significantly (*p* > 0.05). However, nitrite and NOx for L-citrulline + GSH were significantly greater at 30 min post-exercise when compared to placebo (*p* < 0.05).

**Conclusions:**

Combining L-citrulline with GSH augments increases in nitrite and NOx levels during *in vitro* and *in vivo* conditions.

## Introduction

Also known as endothelium-derived relaxing factor (EDRF), nitric oxide (NO) is biosynthesized endogenously from L-arginine and oxygen, by various nitric oxide synthase (NOS) enzymes and by reduction of inorganic nitrate [1]. Cell types containing NOS have been demonstrated to be able to reutilize L-citrulline, the byproduct of NO synthesis, to L-arginine by the arginine-citrulline cycle [2]. Nitric oxide is a gaseous signaling molecule which activates soluble guanylate cyclase (sGC) in smooth muscle cells, thereby catalyzing cyclic guanosine monophosphate (cGMP) synthesis. Intracellular cGMP serves as a cellular messenger and plays a role in a variety of biological processes, and in human blood vessels, results in vasodilation [3]. Cell types containing NOS have been demonstrated to be able to reutilize L-citrulline, the byproduct of NO synthesis, to L-arginine by the arginine-citrulline cycle [2]. An elevation in plasma L-arginine has been shown to improve endothelial function because the vascular endothelium uses NO to signal the surrounding smooth muscle to relax, thus resulting in vasodilation and increasing blood flow [4]. During exercise, vasodilation occurs as a result of various intracellular events, including the production and release of NO. However, it has recently been shown that seven days of oral L-arginine supplementation at 12 g/day, while effective in elevating plasma L-arginine and NO metabolites nitrite and nitrate (NOx) after exercise, was ineffective at increasing blood flow during exercise [5].

L-citrulline has been indicated to be a second NO donor in the NOS-dependent pathway, since it can be converted to L-arginine [6]. Dietary L-citrulline supplementation has shown conflicting results regarding its effectiveness at improving exercise performance [7, 8]. Moreover, results showing favorable effects in exercise performance [8] did not assess NO status; therefore, this response cannot be related to an improvement in exercise performance. The importance of L-citrulline towards ergogenic support is based on the premise that L-citrulline is not subject to pre-systemic elimination and, consequently, could be a more efficient way to elevate extracellular levels of L-arginine. L-Citrulline can perhaps improve the effects on nitrate elimination during the course of recovery from exhaustive muscular exercise, and also serves as an effective precursor of L-arginine. It has been shown that three grams daily of oral L-citrulline supplementation for seven days elevated plasma L-arginine concentration and augmented NO-dependent signaling [9].

Glutathione is a low molecular weight, water-soluble tripeptide composed of the amino acids cysteine, glutamic acid, and glycine. Glutathione is an important antioxidant and plays a major role in the detoxification of endogenous metabolic products, including lipid peroxides. Intracellular glutathione exists in both the oxidized disulfide form (GSSG) or in reduced (GSH) state; the ratio between GSH and GSSG is held in dynamic balance depending on many factors including the tissue of interest, intracellular demand for conjugation reactions, intracellular demand for reducing power, and extracellular demand for reducing potential. In some cell types, GSH appears to be necessary for NO synthesis and NO has been shown to be correlated with intracellular GSH [10]. GSH stimulates total L-arginine turnover and, in the presence of GSH, NOS activity is increased [11]. This suggests that GSH may play an important role in protection against oxidative reaction of NO, thus contributing to the sustained release of NO. Therefore, combining L-citrulline with GSH may augment the production of NO. However, the effectiveness for oral GSH supplementation in humans, particularly in combination with L-citrulline has not been clearly delineated.

Using *in vitro* (cell culture) and *in vivo* approaches in rodents and humans, the overall purpose of this study was to determine the efficacy of L-citrulline and/or GSH supplementation towards increasing the levels of cGMP, nitrite, and NOx. We hypothesized that the combination of L-citrulline and GSH would preferentially increase the concentrations of cGMP, nitrite, and NOx levels when compared to control conditions.

## Methods and procedures

L-citrulline and GSH (Setria®) used in each phase were obtained from KYOWA HAKKO BIO CO., LTD (Tokyo, Japan).

### Phase 1 (*in vitro* efficacy study)

Human umbilical vein endothelial cells (HUVECs) were purchased from Clonetics (San Diego, CA, USA) and cultured in EGM-2 Bullet Kit medium (Clonetics) supplemented with 2 % fetal bovine serum (FBS) and complete endothelial growth factors at 37 ° C in humidified 5 % CO_2_. The cells were seeded into twenty-four well plates 5000 cells/cm2, and sub-confluent cell monolayers were used for experiments. A subset of sub-confluent HUVECs were used as controls and the remainder were treated with either 0.3 mM L-citrulline, 1 mM GSH, or a combination of each at 0.3 mM, and incubated for 24 h. To measure nitrite production by HUVECs, the culture medium was collected and centrifuged to remove any precipitated materials. Four wells for each condition were used and nitrite concentrations of supernatants from each well were determined by high performance liquid chromatography (HPLC) (ENO-20; Eicom, Kyoto, Japan) using our previous approach [12].

### Phase 2 (rodent efficacy study)

This phase of the study was conducted in accordance with the guidelines for the Institutional Animal Care and Use Committee of KYOWA HAKKO BIO CO., LTD. Twenty-three male Sprague–Dawley rats (8 weeks old; Japan SLC, Hamamatsu, Japan) were given free access to standard rat chow (CE-2, CLEA JAPAN Inc., Tokyo, Japan) and tap water in a room with controlled temperature (22 ± 2 ° C), humidity (55 ± 5 %) and a 12-h light/dark cycle. After the rats had been anesthetized with pentobarbital sodium (30 mg/kg, i.p.), a catheter was inserted into the carotid artery. Following 3 days of acclimation, the rats were randomly assigned to 3 groups and received either purified water (CON) (n = 7), L-citrulline (500 mg/kg/day) (n = 8), or a combination of L-citrulline (500 mg/kg/day) plus GSH (50 mg/kg/day) (n = 8) by oral gavage for 3 days. Blood samples were collected from the catheter at baseline and at 0, 0.25, 0.5, 1, 2, and 4 h after the last administration on Day 3. Plasma NOx (nitrite + nitrate) was measured by HPLC (ENO-20; Eicom, Kyoto, Japan) using our previous approach [12].

### Phase 3 (human efficacy study)

#### Participants

Sixty-six apparently healthy, resistance trained [regular, consistent resistance training (i.e., thrice weekly) for at least one year prior to the onset of the study], males between the ages of 18–30 and a body mass index between 18.5–30 kg/m^2^ volunteered to participate in the double-blind, randomized, placebo-controlled, parallel-groups study. Enrollment was open to men of all ethnicities. During the course of the study, six dropped out due to reasons unrelated to the study. As a result, 60 participants completed the study. The age, height, and body mass of participants in each of the four groups can be seen in Table 1. Only participants considered as low risk for cardiovascular disease and with no contraindications to exercise as outlined by the American College of Sports Medicine (ACSM) and who had not consumed any nutritional supplements (excluding multi-vitamins) one month prior to the study were allowed to participate. All participants provided written informed consent and were cleared for participation by passing a mandatory medical screening. All eligible subjects signed university-approved informed consent documents and approval was granted by the Baylor University Institutional Review Board for the Protection of Human Subjects in Research. Additionally, all experimental procedures involved in the study conformed to the ethical consideration of the Declaration of Helsinki.

**Table 1:** Age, height, and body mass of participants in each of the four groups

Group	Age (yrs)	Height (cm)	Body mass (kg)
PLC (n = 15)	21.80 ± 0.92	179.52 ± 2.10	83.92 ± 6.65
GSH (n = 15)	22.67 ± 0.97	179.90 ± 1.71	83.42 ± 2.92
CIT (n = 15)	21.07 ± 0.67	177.17 ± 1.55	80.46 ± 3.17
CIT + GSH (n = 15)	21.67 ± 0.56	179.03 ± 2.34	83.06 ± 2.79

Data are expressed as means ± SEM

#### Entry and familiarization session (visit 1)

Individuals expressing interest in participating in the study were interviewed on the telephone and/or e-mail to determine whether they appeared to qualify to participate in the study. Participants believed to meet eligibility criteria were then invited to attend an entry/familiarization session (visit 1). Once reporting to the lab, individuals were familiarized to the study protocol via a verbal and written explanation outlining the study design and signed an informed consent document. At this point, participants completed a medical history questionnaire and underwent a general physical examination to determine whether they met eligibility criteria. Participants also performed a muscle strength test of the elbow flexors (biceps), and were then given an appointment time to report to the laboratory for a baseline blood sample (visit 2). At this time, participants were instructed to refrain from exercise for 48 h and fast for 8 h prior to baseline blood sampling (visit 2) and post-supplementation testing at day 7 (visit 3).

#### Assessment of elbow flexor muscle strength (visit 1)

In order to determine maximum muscular strength of the elbow flexors, participants performed a one-repetition maximum (1-RM) test on the same elbow flexor machine used in the resistance exercise session based on our previous study [5]. Participants warmed up by completing 5 to 10 repetitions at approximately 50 % of the estimated 1-RM. The participant rested for 1 min, and then completed 3 to 5 repetitions at approximately 70 % of the estimated 1-RM. The weight was then increased conservatively, and the participant attempted to lift the weight for one repetition. If the lift was successful, the participant rested for 2 min before attempting the next weight increment. This procedure was continued until the participant failed to complete the lift. The 1-RM was recorded as the maximum weight that the participant was able to lift for one repetition.

#### Resistance exercise protocol (visit 3, day 7)

Based on our previous study [5], on day 7 participants reported to the Exercise and Biochemical Nutrition Lab at approximately 2:00 pm and performed 3 sets of 15 repetitions with as much weight as they could lift per set (typically 70–75 % of 1RM) involving the elbow flexion exercise on a selectorized weight machine (Body Master, Rayne, LA). Rest periods between sets were timed and lasted exactly 10 s. The resistance exercise session was performed under the direct supervision of study personnel.

#### Venous blood sampling (visit 2, day 0 and visit 3, day 7)

**Fig. 1:**
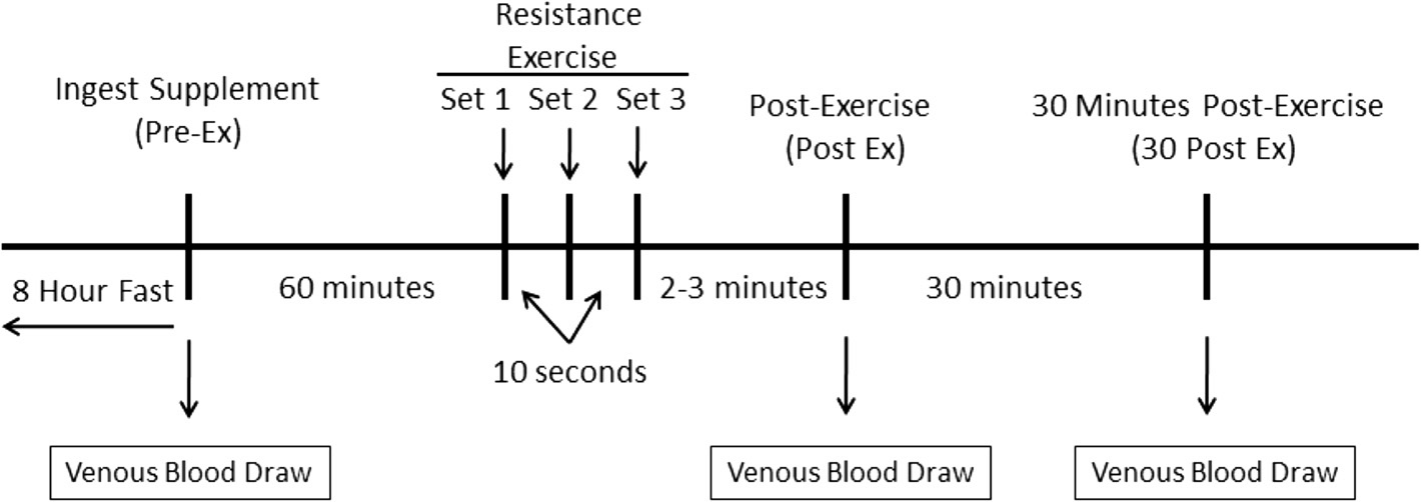
An illustration of the experimental protocol for the testing session at visit 3, following seven days of L-citrulline and/or GSH supplementation

Venous blood samples were obtained from the antecubital vein into 10 ml serum and plasma collection tubes using a standard vacutainer apparatus. Blood samples were allowed to stand at room temperature for 10 min and then centrifuged. The serum and plasma was removed and frozen at −80 °C for later analysis. One baseline blood sample was obtained at visit 2 and 3 samples were obtained at visit 3 (for a total of 4 blood samples). At visit 3 on day 7, the first sample was obtained immediately before ingesting the supplement, the second sample was obtained immediately after resistance exercise, and the third sample 30 min following exercise (Fig. 1).

#### Supplementation protocol

In a randomized, double-blind fashion participants were randomly assigned to one of four groups (n = 15 per group) involving 7 days of the oral ingestion of four capsules containing a total daily dose of either: cellulose placebo (2.52 g/day), L-citrulline (2 g/day), GSH (1 g/day), or L-citrulline (2 g/day) + GSH (200 mg/day). The total weight of the four capsules for each group was the same. Each participant ingested all four capsules containing their respective daily supplement dose each evening for six consecutive days. At Visit 3 (Day 7), participants were provided the final daily dose of their respective supplement ingested one hour prior to performing the resistance exercise. Supplementation compliance was monitored by participants returning empty containers of their supplement on day 7, and also by completing a supplement compliance questionnaire.

#### Assessment of blood variables (L-citrulline and L-arginine)

To determine plasma L-citrulline and L-arginine concentrations, the plasma was de-proteinated by mixing equal volumes of plasma and trichloroacetic acid (TCA) (6.0 % wt/vol). The samples were vortexed and centrifuged for 15 min at 12,000 × g. Amino acids in the supernatant were analyzed with an amino acid analyzer (L-8900, Hitachi, Japan).

#### Assessment of plasma cGMP and nitrite

From the blood samples obtained at visit 2 (day 0) and visit 3 (day 7), using commercially-available enzyme-linked immunoabsorbent assay (ELISA) kits (Cayman Chemical, Ann Arbor, MI, USA), plasma cGMP, nitrite, and NOx were determined. Assays were analyzed in duplicate and absorbances for each variable were determined at a wavelength of 450 nm using a microplate reader (iMark, Bio-Rad, Hercules, CA). A set of standards of known concentrations for each variable utilized to construct standard curves and concentrations were determined using data reduction software (Microplate Manager, Bio-Rad, Hercules, CA).

### Statistical analysis

For *in vitro* (phase 1), rodent (phase 2), and the human (phase 3) efficacy studies, results were expressed as mean ± SEM. Delta values (differences between the baseline and sequential values) were analyzed using Bonferroni’s test following one-way ANOVA. For multiple comparisons to identify the statistical differences among treatments, the Bonferroni correction or Dunnett’s multiple test following a comparison of the data by non-repeated ANOVA was employed. Statistical significance was considered as a p-value ≤ 0.05. Statistical analysis was performed using Statcel software for Windows (Version 2, OMS Publishing, Inc., Saitama, Japan) and the Systat 2000 Statistical Program File (Igaku Tosho Shuppan, Tokyo, Japan).

## Results

### Phase 1 (*in-vitro* cell culture study)

**Fig. 2:**
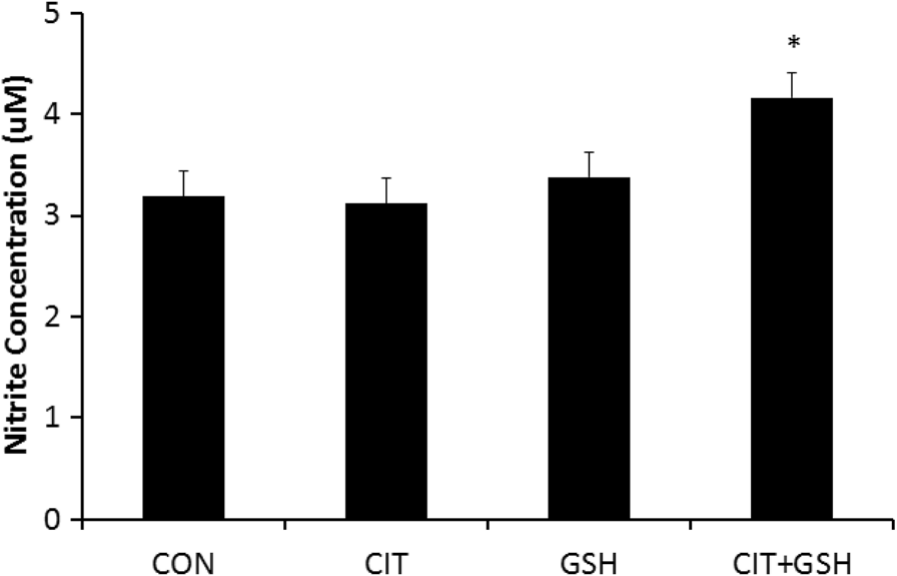
From phase one, an illustration of nitrite concentration in HUVECs following supplementation with L-citrulline and/or GSH. The symbol * indicates that cells supplemented with a combination of L-citrulline (CIT) and GSH underwent significant increases in nitrite formation compared to cells supplemented with phosphate buffered saline (CON) (*p* < 0.05)

Results demonstrated no significant differences between the control condition and cells treated with L-citrulline and GSH (*p* > 0.05) for nitrite concentration. However, cells treated with L-citrulline and GSH were significantly greater than control-treated cells (*p* < 0.05) (Fig. 2).

### Phase 2 (rodent efficacy study)

**Fig. 3:**
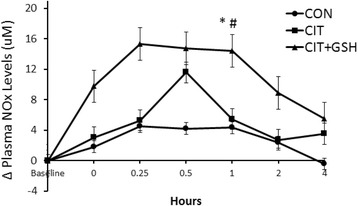
From phase two, an illustration of plasma NOx levels in rats following supplementation with L-citrulline and/or GSH. The symbols * and ^#^ indicate that combined L-citrulline (CIT) and GSH-supplemented rats underwent significant increases in plasma NOx at one hr following supplement infusion compared to animals supplemented with water (CON) and CIT, respectively (*p* < 0.05)

For plasma NOx delta values, results demonstrated that L-citrulline + GSH was significantly greater than control and L-citrulline at one hr post-supplement infusion (*p* < 0.05) (Fig. 3).

### Phase 3 (human efficacy study)

#### Plasma L-arginine and L-citrulline

**Fig. 4:**
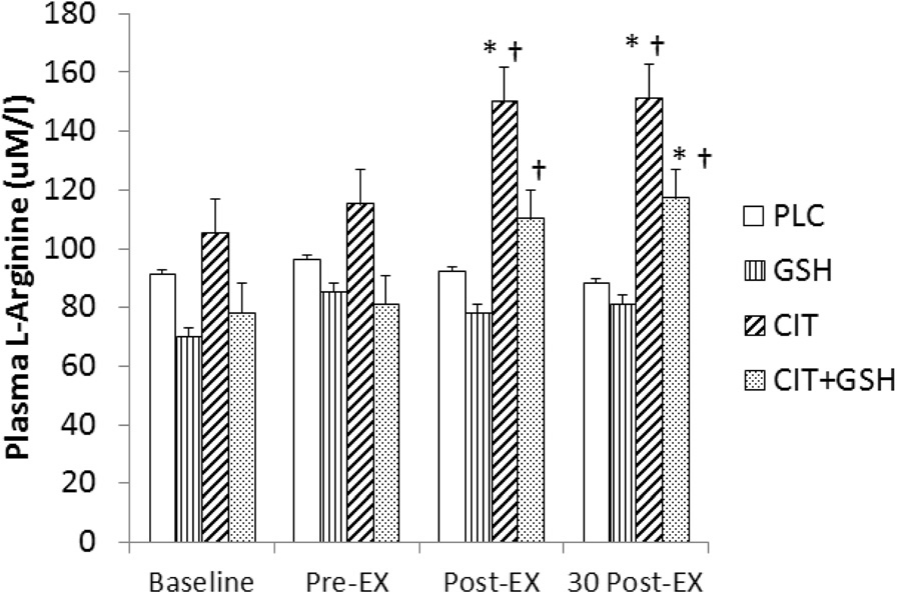
From phase three, an illustration of plasma arginine levels in humans following seven days of supplementation with L-citrulline and/or GSH. Results indicated that L-citrulline (CIT) and a combination of CIT + GSH produced significant increases in plasma arginine immediately after and 30 min post-exercise compared to groups supplemented with cellulose (PLC) and GSH. The symbol * indicates a significant increase compared to PLC and the symbol ^†^ indicates a significant increase compared to GSH (*p* < 0.05)

**Fig. 5:**
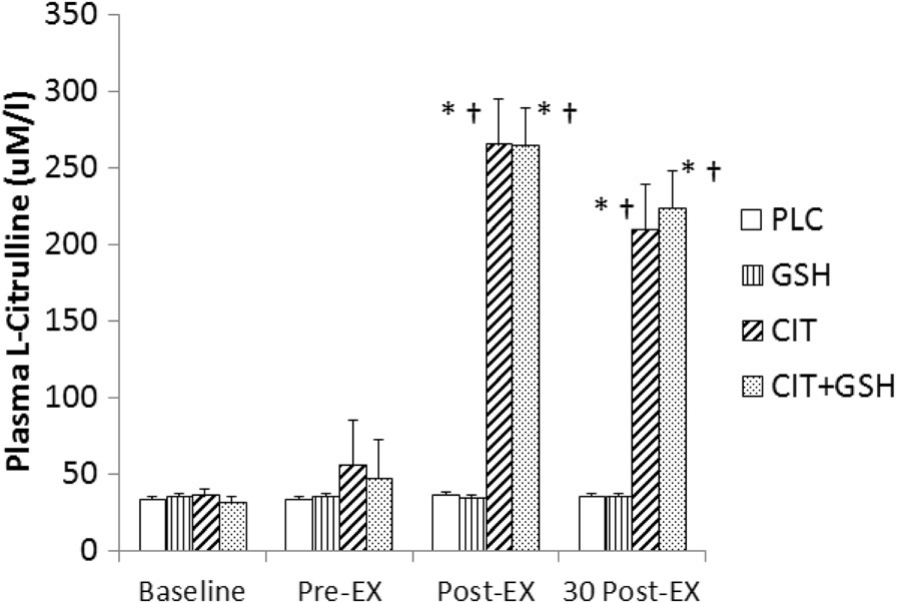
From phase three, an illustration of plasma citrulline levels in humans following seven days of supplementation with L-citrulline and/or GSH. Results indicated that L-citrulline (CIT) and a combination of CIT + GSH produced significant increases in plasma CIT immediately after and 30 min post-exercise compared to groups supplemented with cellulose (PLC) and GSH. The symbol * indicates a significant increase compared to PLC and the symbol ^†^ indicates a significant increase compared to GSH (*p* < 0.05)

Since no supplementation was involved at the baseline testing session, as expected, no significant differences between groups or time points (*p* > 0.05) for plasma L-citrulline and L-arginine were observed. However, at the follow-up testing session following seven 7 days of supplementation significant increases for plasma L-arginine and L-citrulline were noted. For L-arginine, no significant differences occurred between placebo and GSH at any time points (*p* > 0.05). However, at the immediate post-exercise time point L-citrulline was significantly greater than placebo and GSH, whereas L-citrulline + GSH was greater than GSH (*p* < 0.05). In addition, at 30 min post-exercise L-citrulline and L-citrulline + GSH were both significantly greater than placebo and GSH (*p* < 0.05) (Fig. 4). For plasma L-citrulline, L-citrulline and L-citrulline + GSH were both significantly greater than placebo and GSH immediately post-exercise and at 30 min post-exercise (*p* < 0.05) (Fig. 5).

#### Plasma cGMP, nitrite, and NOx

**Fig. 6:**
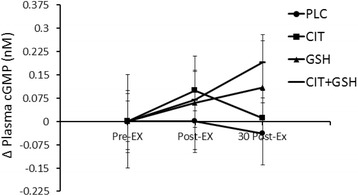
From phase three, an illustration of plasma cGMP levels in humans following seven days of supplementation with L-citrulline and/or GSH. Results indicated no significant differences between any of the groups at any of the assessed time points (*p* > 0.05)

**Fig. 7:**
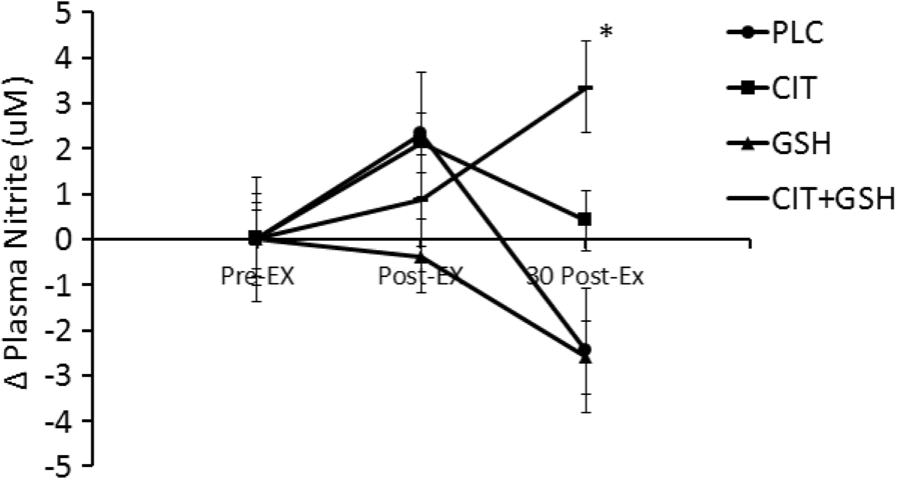
From phase three, an illustration of plasma nitrite levels in humans following seven days of supplementation with L-citrulline and/or GSH. The symbol * indicates that a combination of L-citrulline (CIT) and GSH produced significant increases in plasma nitrite at 30 min post-exercise compared to the group supplemented with cellulose placebo (PLC) (*p* < 0.05)

**Fig. 8:**
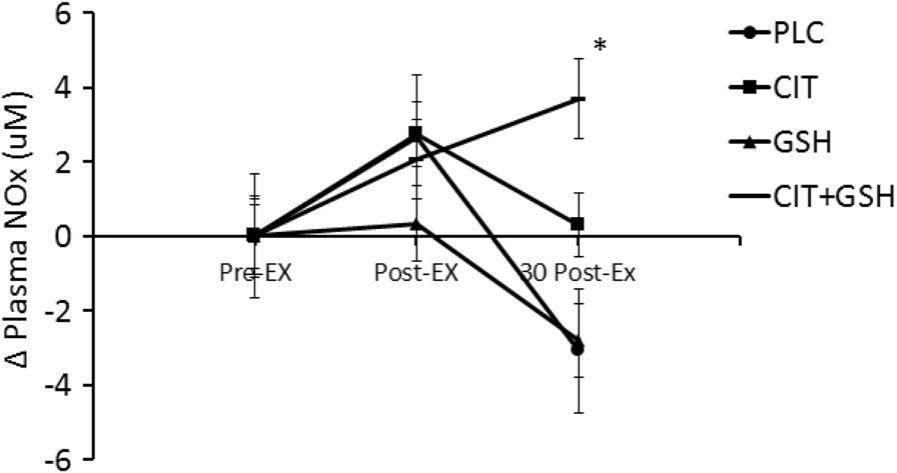
From phase three, an illustration of plasma NOx levels in humans following seven days of supplementation with L-citrulline and/or GSH. The symbol * indicates that a combination of L-citrulline (CIT) and GSH produced significant increases in plasma NOx at 30 min post-exercise compared to the group supplemented with cellulose placebo (PLC) (*p* < 0.05)

The delta values for the plasma levels of cGMP, nitrite, and NOx can be seen in Figs. 6, 7 and 8, respectively. For cGMP (Fig. 6), L-citrulline + GSH was elevated compared to the other three groups, but there were no significant differences between groups and time points observed (*P* > 0.05). For nitrite (Fig. 7) and NOx (Fig. 8), L-citrulline + GSH was significantly greater than placebo at 30 min post-exercise (*P* < 0.05).

## Discussion

In the present study, we sought to determine the effectiveness of L-citrulline and/or GSH in increasing NO synthesis during *in vivo* conditions with rodents and humans and also in an *in vitro* condition using HUVEC. Collectively, in phase one and three of the study we observed combining L-citrulline with GSH to be more effective at increasing the concentrations of nitrite and/or NOx than with control/placebo in HUVEC and humans, respectively. In phase two, we observed L-citrulline combined with GSH to be more effective at increasing plasma NOx.

L-citrulline is a ubiquitous amino acid in mammals [13], and in the kidneys, vascular endothelium, and other tissues can be readily converted to L-arginine thus raising plasma and tissue levels of L-arginine which increases NOS synthesis and subsequent NO production [14]. Additionally, L-citrulline has been indicated to be a secondary NO donor in the NOS-dependent pathway, since it can be converted to L-arginine. Nitrate and nitrite are the main substrates to produce NO via the NOS-independent pathway. These anions can be reduced *in vivo* to NO and other bioactive nitrogen oxides.

Previous studies have reported that L-citrulline could increase plasma L-arginine concentration by the L-citrulline-NO cycle [15]. Fu et al. [16] showed that pre-treatment with L-citrulline in rodents for seven days at doses of 300, 600, and 900 mg/kg increased the NO content. Since L-citrulline can be readily converted to L-arginine, it provides a recycling pathway for the conversion of L-citrulline to NO via L-arginine [14, 17]. In phase three of the present study, we observed seven days of L-citrulline supplementation, with and without GSH, to result in significant increases in the levels of plasma citrulline and arginine. Our present data support previous results [18] showing that a 10-g oral bolus of L-citrulline significantly enhanced plasma citrulline and arginine levels compared with placebo. Therefore, our present observations indicate that L-citrulline is indeed a precursor to L-arginine formation which subsequently increases circulating levels of NOx, and that recycling of L-citrulline to L-arginine may maintain substrate concentration in favor of NO synthesis [19].

It has been shown in some mammalian cell types, that GSH and NO activity are linked [20]. Furthermore, results suggest that GSH is necessary in HUVEC for NO synthesis rather than for the NO-related effect on guanylate cyclase, because when cells were depleted of GSH, citrulline synthesis and cGMP production were inhibited in a concentration-dependent manner [21]. This may be explained based on the premise that the synthesis of NO, detected as L-citrulline production, in HUVEC and murine endothelial cells has been shown to be correlated with intracellular GSH [10]. A previous study suggested that in some cell types, the activity of NO is influenced by the endogenous antioxidant GSH [22]. It is conceivable that GSH activity may be augmented by L-citrulline as it has been shown that pre-treatment with L-citrulline in rodents for seven consecutive days lead to an elevation in the level of GSH [23].

Furthermore, in phase one of the present study, we showed that combining L-citrulline and GSH effectively increased nitrite concentration in HUVEC cells compared to control; although, both L-citrulline and GSH alone had no effect on nitrite. However, in phase two, the combined L-citrulline and GSH provided to rodents resulted in a significant increase in plasma NOx one hr following ingestion compared to control and L-citrulline. Moreover, we observed a similar response in phase three compared to phase one, where combining L-citrulline and GSH effectively increased plasma nitrite and NOx concentration in humans compared to placebo.

Oral supplementation with L-arginine can increase plasma L-arginine levels; although, oral supplementation with L-citrulline, a precursor for arginine biosynthesis, has been shown to be more efficient than oral L-arginine in increasing plasma L-arginine [9], due to splanchnic catabolism of ingested L-Arginine [24]. NO synthesis is primarily dependent upon intracellular arginine availability and is affected by: 1) the transport of extracellular arginine; 2) intracellular synthesis of arginine from citrulline, which is dependent on citrulline availability; and 3) the activity of arginase [17]. Moreover, this latter point can be further supported based on the data demonstrating increased arginine availability in cultured cell model or by supplementation *in vivo* was able to overcome the effects of arginase and to enhance NO synthesis [25]. Based on results from all three phases of the present study, it is evident that L-citrulline supplementation impacted extracellular arginine concentration and the subsequent intracellular arginine synthesis based on the responses we observed in nitrite and NOx concentrations.

In phase 3 of the present study, we were also interested to determine if increased plasma arginine availability and subsequent NO synthesis due to oral L-citrulline and/or GSH supplementation was effected by resistance exercise. Interestingly, we observed increases in plasma NOx in all four groups immediately following resistance exercise, which indicates this response in plasma NOx to be particularly due to the stimulus of resistance exercise. These results are similar to our previous study where resistance exercise increased plasma NOx, independent of increased plasma arginine, due to seven days of L-arginine supplementation [5]. In the same way as NOx, plasma cGMP levels were increased by the combination of L-citrulline and GSH; however, this increase was not significantly different. Nevertheless, this suggests a possible synergistic effect from GSH that may be partially mediated by the formation of the NO-GSH complex. However, in the present study, significantly different increases in NOx occurred 30 min following resistance exercise, and only for the L-citrulline + GSH group. This suggests that a resistance exercise-related mechanism of inducing plasma NO, perhaps due to increased shear stress that triggered an up-regulation in NO-cGMP signaling, is a conceivable candidate for this response.

Consequently, there are possible physiological benefits of having high NO levels at 30 min post-exercise relative to its impact on muscle protein metabolism and possible muscle performance in response to resistance exercise training. It has been shown that NOS activity is necessary for calcium-induced activation of the Akt pathway (involved in translation initiation and thus muscle protein synthesis), and that NO is sufficient to elevate Akt activity in primary myotubes. Nitric oxide appears to influence Akt signaling though a cGMP/PI3K-dependent pathway [26], which is the primary pathway for up-regulating translation initiation and protein synthesis in skeletal muscle. Additionally, nitrite has been shown to enhance the proliferation and mTOR activity of myoblasts [27]. Similarly, NO seems to influence skeletal muscle function through effects on excitation-contraction coupling, myofibrillar function, perfusion, and metabolism. Another study showed that by using an agent to inhibit phosphodiesterase-5, that the augmentation of NO-cGMP signaling increased protein synthesis and reduced fatigue in human skeletal muscle [28]. In the present study, L-citrulline + GSH showed an improvement in cGMP activity suggesting that this outcome could likely play a role in muscle protein synthesis and muscle performance when combined with resistance training.

Our present data suggest that the oral supplementation of L-citrulline combined with GSH provides an augmenting effect on plasma NOx. Based on results from recent studies, this may be explained based on the premise that in some cell types, the activity of NO is influenced by the endogenous antioxidant, GSH [10]. Therefore, GSH may play an important role in protection against oxidative reaction of NO, thus contributing to the sustained release of NO.

## Conclusions

Herein, we have presented *in vitro* and *in vivo* data demonstrating the efficacy of combining L-citrulline and GSH and the subsequent effects on NO synthesis and, collectively, we conclude that the combination of L-citrulline and GSH increases the levels of cGMP, nitrite, and NOx.
